# Islet autoantibody seroconversion in type-1 diabetes is associated with metagenome-assembled genomes in infant gut microbiomes

**DOI:** 10.1038/s41467-022-31227-1

**Published:** 2022-06-21

**Authors:** Li Zhang, Karen R. Jonscher, Zuyuan Zhang, Yi Xiong, Ryan S. Mueller, Jacob E. Friedman, Chongle Pan

**Affiliations:** 1 Harold Hamm Diabetes Center, University of Oklahoma Health Sciences Center, Oklahoma City, OK USA; 2 Department of Microbiology and Plant Biology, University of Oklahoma, Norman, OK USA; 3 Department of Biochemistry and Molecular Biology, University of Oklahoma Health Sciences Center, Oklahoma City, OK USA; 4 School of Computer Science, University of Oklahoma, Norman, OK USA; 5 Department of Microbiology, Oregon State University, Corvallis, OR USA; 6 Department of Physiology, University of Oklahoma Health Sciences Center, Oklahoma City, OK USA

**Keywords:** Metagenomics, Type 1 diabetes

## Abstract

The immune system of some genetically susceptible children can be triggered by certain environmental factors to produce islet autoantibodies (IA) against pancreatic β cells, which greatly increases their risk for Type-1 diabetes. An environmental factor under active investigation is the gut microbiome due to its important role in immune system education. Here, we study gut metagenomes that are *de-novo*-assembled in 887 at-risk children in the Environmental Determinants of Diabetes in the Young (TEDDY) project. Our results reveal a small set of core protein families, present in >50% of the subjects, which account for 64% of the sequencing reads. Time-series binning generates 21,536 high-quality metagenome-assembled genomes (MAGs) from 883 species, including 176 species that hitherto have no MAG representation in previous comprehensive human microbiome surveys. IA seroconversion is positively associated with 2373 MAGs and negatively with 1549 MAGs. Comparative genomics analysis identifies lipopolysaccharides biosynthesis in *Bacteroides* MAGs and sulfate reduction in *Anaerostipes* MAGs as functional signatures of MAGs with positive IA-association. The functional signatures in the MAGs with negative IA-association include carbohydrate degradation in lactic acid bacteria MAGs and nitrate reduction in *Escherichia* MAGs. Overall, our results show a distinct set of gut microorganisms associated with IA seroconversion and uncovered the functional genomics signatures of these IA-associated microorganisms

## Abstract (web summary)

Here, by characterizing gut metagenomes of at-risk children in the TEDDY project, the authors associate onset of autoimmunity leading to Type-1 diabetes with certain sets of microorganisms in the gut microbiota, and identify metabolic capabilities encoded in the genomes of these microorganisms that provide functional insights to the association.

## Introduction

Type 1 diabetes (T1D) is an autoimmune disease that often manifests during childhood and adolescence and is characterized by insulin deficiency resulting from destruction of pancreatic β cells^1^ Over 2/3 of children with seroconversion to multiple islet autoantibodies (IA) progress to T1D within 10 years^2,3^ IA seroconversion is characterized by the presence of autoantibodies to antigens of pancreatic β cells, including insulin (IAA), glutamic acid decarboxylase (GADA), insulinoma-associated autoantigen 2 (IA2A), and/or zinc transporter 8 (ZnT8A)^2^ The risk of developing islet autoimmunity declines with age, and the influence of major genetic factors on this risk is limited to the first few years of life^4^

Increasing evidence shows that environmental factors play an important role in the onset of IA autoimmunity and the progression of T1D in children and young adults^5–8^ The gut microbiota is a key environmental factor that interacts with the immune system to trigger IA seroconversion or T1D pathogenesis^6–9^ A number of longitudinal studies, including TEDDY^6,10^, DIABIMMUNE^11^, FINDIA^12^ and ABIS^13^, characterized the role of the gut microbiota in the development of autoimmunity using large, at-risk cohorts. These studies found that the fecal microbiota of individuals with IA seroconversion or recent T1D onset display a lower taxonomic diversity, with a larger representation of *Bacteriodetes*, than those of control individuals, although the association with specific taxa varied among the studies^7,8,11^

The Environmental Determinants of Diabetes in the Young (TEDDY) study was designed to identify the environmental risk factors for T1D by monitoring children at high genetic risk for development of T1D. In the TEDDY study, fecal samples were collected monthly from 887 subjects, beginning at 3 months of age. A total of 12,276 fecal microbiomes were sequenced using both 16 S rRNA amplicon sequencing and metagenomic shotgun sequencing strategies. Analysis of the 16 S sequence data^10^ revealed subtle, but significant, changes in the relative abundances of bacterial species’ 16 S gene copies between IA or T1D cases and controls. 16 S genes from an unclassified *Erysipelotrichaceae* were more prevalent in IA cases than in controls. T1D cases had a higher abundance of *Parabacteroides* and lower abundances of 11 genera, including four unclassified *Ruminococcaceae*, *Lactococcus*, *Streptococcus*, and *Akkermansia*, than controls. Analyses of the metagenomic data in Vatanen, et al.^6^ found that IA cases had a higher prevalence of metagenome reads assigned as *Streptococcus* group *mitis/oralis/pneumoniae*, while controls had higher abundances of reads from *Lactobacillus rhamnosus* and *Bifidobacterium dentium*. T1D cases had a higher abundance of reads from *Bifidobacterium pseudocatenulatum*, *Roseburia hominis*, and *Alistipes shahii*, while controls had a higher prevalence of *Streptococcus thermophilus* and *Lactococcus lactis* reads. Comparison of gene abundances and pathway analysis supported protective effects of short-chain fatty acids against T1D^6^ Overall, these studies revealed weak associations between T1D and IA seroconversion and several bacterial taxa.

Metagenomic data from the TEDDY study were analyzed in Stewart, et al.^10^ and Vatanen, et al.^6^ using a two-step read-mapping approach. In the first step, the taxonomic composition of a metagenome was estimated with MetaPhlAn2^14^ by mapping reads onto a database of clade-specific marker genes. In the second step, the functional profile of a metagenome was inferred with HUMAnN2^15^ by mapping reads onto selected reference genomes and UniRef90. However, many human gut microorganisms are not represented by reference genomes and large protein sequence spaces are not captured in protein databases^16–18^ This creates large “blind spots” when applying the read-mapping approach, because short reads from undescribed microorganisms and divergent protein-coding genes cannot be confidently mapped and, therefore, cannot be accounted for in subsequent statistical comparisons.

Here, we use a genome-resolved metagenomics approach to re-analyze the TEDDY microbiome data to accomplish the following objectives. First, we aimed to determine whether metagenome assembly and binning can be effectively achieved in a large-scale longitudinal microbiome study. While this approach has been carried out in small-scale longitudinal microbiome studies^19,20^, here we used data from the TEDDY study and demonstrated that this approach is scalable to hundreds of subjects over multiple years. A total of 21,536 high-quality metagenome-assembled genomes (MAGs) were obtained from TEDDY cohort data, including 176 previously undescribed human microbiome species, which further expand the diversity of human MAG collections^18,21,22^ Second, we aimed to test the hypothesis that a core microbiome within the TEDDY microbiomes can be identified from metagenome assemblies based on protein families. These protein families in the core microbiome are represented in the majority of subjects and can account for the majority of the microbiome genetic content. Third, while Stewart, et al.^10^ and Vatanen, et al.^6^ have investigated the association of bacterial lineages or functions with IA seroconversion, we aimed to identify MAGs significantly associated with IA seroconversion. These MAGs were compared with background MAGs in adjacent lineages to identify metabolic pathways over-represented in the MAGs with IA association.

## Results

### Longitudinal binning of metagenome-assembled genomes

The TEDDY project shotgun-sequenced the metagenomes of 12,276 fecal samples donated by a cohort of 887 subjects when they were between 3 and 72 months of age^6,10^ We obtained the metagenomic sequencing data from the dbGap database. All fecal samples from the same subject were combined and co-assembled into a composite metagenome for each respective subject. The 887 subject-specific metagenomes containing scaffolds larger than 2 kbps had a median size of 142 million base pairs (Mbps) with a first-quartile (Q1) size of 78 Mbps and a third-quartile (Q3) size of 221 Mbps. L50 is the scaffold length threshold above which longer scaffolds add up to 50% of the total metagenome size, and the median L50 of the metagenomes was 15,244 bps (Q1 = 12,736 bp and Q3 = 18,944 bp). Importantly, the median mapping rate of the reads from 12,276 fecal samples to each respective assembly was 89% (Q1 = 85% and Q3 = 92%). The high percentage of read mapping to each assembly indicates that high-quality and near-complete metagenome assemblies, representing the majority of microbial populations within the fecal microbiomes, were obtained for most subjects across the developmental stages surveyed by this study (Supplementary Data 1).

The relative abundances of all scaffolds in an individual fecal sample were estimated by mapping reads from each fecal sample onto the respective composite metagenome assembly of the subject from whom the fecal sample was obtained. These coverage data represented the longitudinal abundance profiles of every scaffold for each subject, which were used for binning MAGs from each subject’s composite metagenome. From 887 subject-specific composite metagenomes, 21,536 high-quality MAGs (completeness > 90%; contamination < 5%) and 15,796 medium-quality MAGs (completeness > = 50%; contamination <10%) were obtained (Supplementary Fig 1; Supplementary Data 2). Quality assessment was based on the minimum information about a metagenome-assembled genome (MIMAG) standard^23^ Assembly statistics of the MAGs in this study are summarized in Supplementary Fig 1. The median mapping rate of a subject’s time-specific metagenomic reads onto all of the high-quality MAGs generated from the respective composite metagenome is 51% (Q1 = 37% and Q3 = 65%). Thus, the high-quality MAGs represented common microbial populations in many fecal samples. For brevity, we focus on only the high-quality MAGs below, unless otherwise noted.

**Fig. 1:**
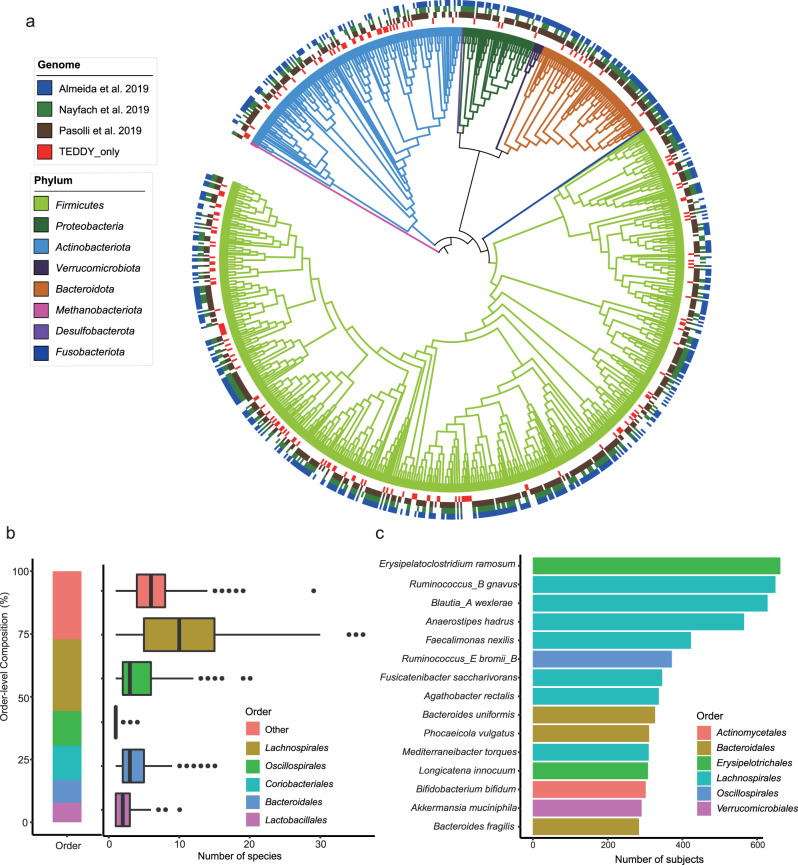
Phylogeny and taxonomy distribution of high-quality MAGs from TEDDY. **a** Taxonomy tree of the 883 species represented by TEDDY MAGs. Branches are colored at the phylum level. The four rings mark the 567 species matched to Almeida, et al.^21^ in blue, the 458 species matched to Nayfach, et al.^18^ in green, the 626 species matched to Pasolli, et al.^22^ in brown, and the 176 species only identified from TEDDY in red. **b** Order-level composition of the 883 TEDDY species (left) and the number of species per subject in each order (right). Only the five most common orders, *Lachnospirales* (*n* = 823 subjects), *Oscillospirales* (*n* = 648), *Coriobacteriales* (*n* = 321), *Bacteroidales* (*n* = 698), and *Lactobacillales* (*n* = 615), are shown individually, while the remaining orders are grouped as ‘other’ (*n* = 3752). Most subjects had less than 10 species from each of the orders except *Lachnospirales*. Boxplots show the median (center), the first and third quartile (bounds of box), and 1.5X interquartile ranges (whiskers). Points beyond the ends of whiskers are outliers. **c** Species identified in the largest numbers of subjects in TEDDY, colored by orders. Source data are provided as a Source Data file.

21,276 of the 21,536 high-quality MAGs were assigned to taxonomies of 706 bacterial reference species and two archaeal reference species in the Genome Taxonomy Database (GTDB). The remaining 260 high-quality MAGs were clustered, based on 95% average nucleotide identity (ANI) between scaffolds of each MAG, into 175 distinct metagenomic species not found within the GTDB. In total, the high-quality MAGs represented 883 distinct species (Fig. 1a and Supplementary Data 3). The five most prevalent orders were *Lachnospirales* (*n* = 253 species), *Oscillospirales* (*n* = 121), *Coriobacteriales* (*n* = 120), *Bacteroidales* (*n* = 81) and *Lactobacillales* (*n* = 69) (Fig. 1b). On average, the high-quality MAGs from a given subject’s composite metagenome represented 24 species. The 15 most prevalent species identified in this study, across the 887 subjects, are shown in Fig. 1c. Four species in the Firmicutes phylum were widely distributed across individuals and were present in more than half of the composite metagenomes of all subjects (Fig. 1c). These species include *Erysipelatoclostridium ramosum* (*n* = 662 subjects), *Ruminococcus_B gnavus* (n = 649), *Blautia_A wexlerae* (*n* = 628) and *Anaerostipes hadrus* (*n* = 565).

Species-level assignments were compared with three previous large-scale surveys of MAGs in human gut microbiota^18,21,22^ Almeida, et al.^21^ recovered 39,891 high-quality MAGs from 13,133 human gut metagenomic datasets from 75 different studies. Nayfach, et al.^18^ recovered 60,664 MAGs from 3,810 human gut metagenomic datasets, which were clustered to 2,935 species together with reference genomes from PATRIC^24^ and IMG^25^ Pasolli, et al.^22^ recovered 154,723 microbial genomes (70,178 high quality) from 9,428 metagenomes spanning body sites, ages, countries, and lifestyles. In general, taxonomic distributions of the MAGs were consistent between our study and the three previous studies. Of the 883 species recovered in our MAG analysis of the TEDDY data, 567 species matched to Almeida, et al.^21^, 458 matched to Nayfach, et al.^18^, 626 matched to Pasolli, et al.^22^, and 707 matched to their union (Fig. 1a). A total of 176 species represented by 356 MAGs were not recovered in any of these existing datasets, nor were these species found in the reference genomes of human gut microbiomes from the PATRIC and IMG databases. Therefore, we recovered 356 high-quality MAGs for 176 previously undescribed species in human gut microbiomes (Supplementary Data 3). These species further expand the taxonomic range of microbial genomes found to inhabit the human gut.

**Fig. 2:**
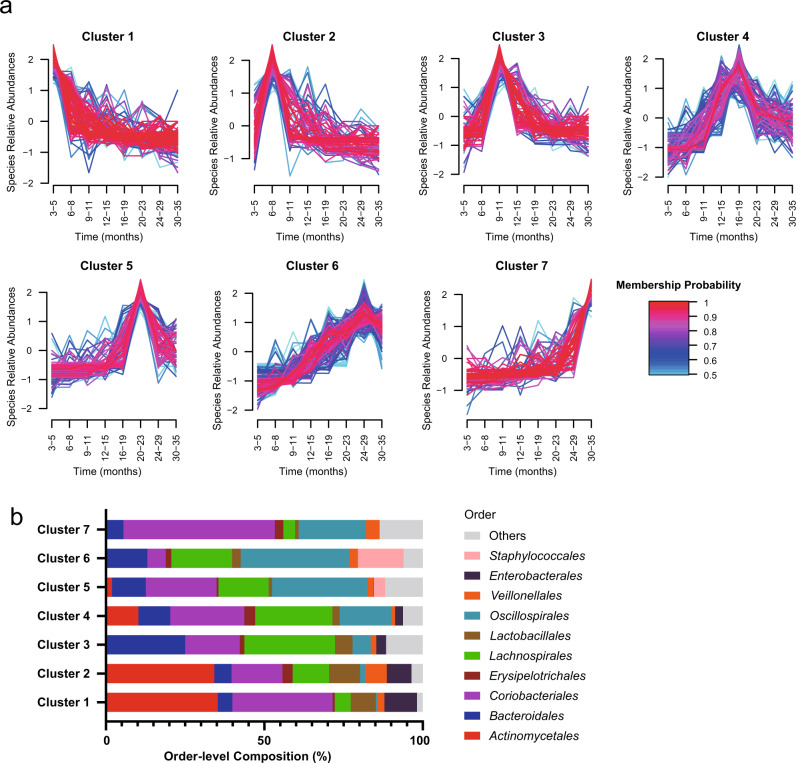
Genome-resolved longitudinal abundance profiles of TEDDY microbiomes. **a** Species clustered into seven groups by their average MAG abundances across eight developmental stages, shown in months on the x-axis. Clusters are ordered temporally by their peak abundances in the developmental stages from month 3 to month 35. Line colors indicate the membership probabilities of the species. Only species with a membership probability greater than 0.5 are shown. Abundances were standardized to a mean value of zero and a standard deviation of one. **b** Order-level composition of species identified in the seven clusters. Source data are provided as a Source Data file.

The TEDDY metagenome project provided a genome-resolved longitudinal profile of gut bacterial development. We evaluated species abundance profiles across eight time-periods, including 3 to 5 months of age, 6 to 8 months of age, 9 to 11 months of age, 12 to 15 months of age, 16 to 19 months of age, 20 to 23 months of age, 24 to 29 months of age, and 30 to 35 months of age. The 883 species were clustered into seven groups with similar profiles of temporal abundance changes (Fig. 2a and Supplementary Data 4). The seven clusters all featured a single peak of species abundance in different time periods: months 3–5 for cluster 1, months 6–8 for cluster 2, months 9–11 for cluster 3, months 16–19 for cluster 4, months 20–23 for cluster 5, months 24–29 for cluster 6, and months 30–35 for cluster 7 (Fig. 2a). Each cluster was comprised of distinct groups of microorganisms (*p*-value = 1.23E-17, Chi-square test, two-sided), shown at the order level in Fig. 2b. For example, the abundance of *Actinomycetales* and *Enterobacterales* were higher in cluster 1–2 than the other clusters (*p*-value = 1.90E-4 and 1.92E-3, respectively, Student’s *t*-test, two-sided), the abundance of *Lactobacillales* was higher in clusters 1–3 (*p*-value = 0.003, Student’s *t*-test, two-sided), and *Oscillospirales* had increased abundances in clusters 4–7 compared with clusters 1–3 (*p*-value = 0.007, Student’s *t*-test, two-sided).

### Core protein families in childhood gut microbiota

**Fig. 3:**
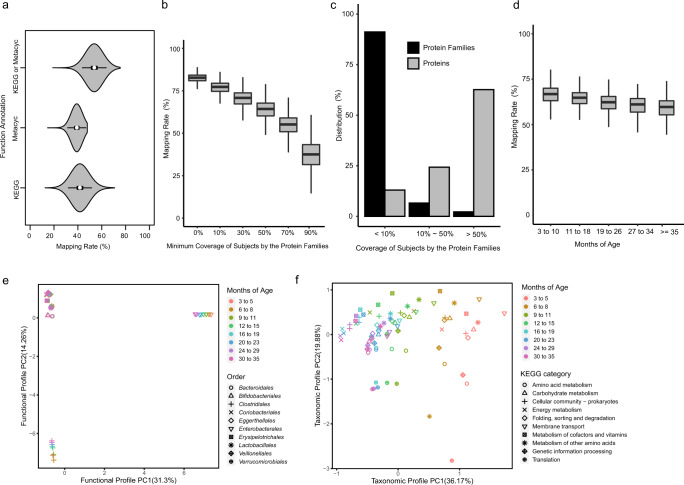
Construction of protein core families from human gut microbiota. **a** Mapping rates of metagenomic reads onto protein-coding genes with functional annotations. Violin plots show the distributions of annotation mapping rates across samples (*n* = 12,854 metagenomic sequencing runs). Over half of the genetic potential in a sample have functional annotations from MetaCyc or KEGG. Violin plots indicate median (white dot), the first and third quartile (black bar in the center), and the 1.5X interquartile ranges (black lines stretched from the bar). **b** Mapping rates of metagenomic reads onto protein families present in more than certain percentages of subjects in each metagenomic sequencing run (*n* = 12,854). All protein families (0% on the x-axis) accounted for 82.4% of the metagenomic reads. The core protein families were defined to be families found in >50% of subjects (50% on the x-axis), which accounted for 63.6% of the metagenomic reads. **c** Distribution of proteins across protein families. The core protein families in >50% of subjects represented 2.2% of all families, but included 62.7% of all proteins. The peripheral protein families in less than 10% of subjects represented 91.2% of all families, but included only 13% of all proteins. **d** Mapping rates of metagenomic reads onto the core protein families across the following developmental stages defined by the months of age: 3 to 10 (*n* = 4,645), 11 to 18 (*n* = 3,634), 19 to 26 (*n* = 2,252), 27 to 34 (*n* = 1,385), and ≥35 (*n* = 938). The mapping rates only decreased slightly as the subjects matured and their microbiomes diversified. **e** Principal component analysis (PCA) of the functional profiles of major orders over time. The functional profile of an order is the gene abundances of core families in this order in every KEGG category. **f** PCA of the taxonomic profiles of KEGG categories over time. The taxonomic profile of a KEGG category is the gene abundances of core families in this KEGG category in every order. Boxplots show the median (center), the first and third quartile (box), and 1.5X interquartile ranges (whiskers). Source data are provided as a Source Data file.

A total of ~156 million protein-coding sequences were predicted from the metagenome assemblies. On average, function annotation can be assigned to 40% of the proteins by KEGG Orthology (KO) terms, 37% by MetaCyc reactions, and 54% by at least one of the two annotation systems (Supplementary Fig 2a). Genes encoding proteins with KEGG, MetaCyc, or either annotation accounted for 42%, 39%, and 54% of the reads from an average metagenome, respectively (Fig. 3a). This assembly and gene prediction approach produced a more comprehensive annotation profile than read-based annotations, as less than 10% of the metagenomic reads had MetaCyc annotation when this dataset was analyzed using a read mapping approach^6^

Predicted protein-coding sequences from all metagenomes were clustered into 2,885,868 homologous protein families. ~91% of the families were found in <10% subjects, ~7% in 10%~50% subjects, and ~2% in >50% subjects (Fig. 3c and Supplementary Fig 2b). The 64,142 protein families (~2% of all families) that were present in >50% subject metagenomes contained 63% of predicted proteins encoded by these metagenomes and accounted for 64% of the reads on average (Fig. 3b, c; Supplementary Data 5). The 50 most frequent Enzyme Commission (E.C.) number annotations of the protein families and their genus-level taxonomic distributions are shown in Supplementary Fig 2c. The read mapping rate to these 64,142 conserved and widely-distributed protein families decreased from 67% to 60% over the time course of the TEDDY study (Fig. 3d). These findings indicate that the proteome of each fecal microbiome is comprised of a few well-conserved and widely-distributed core protein families coupled with many rare and poorly conserved families. The core protein families have functional annotations enriched in fundamental cellular functions, such as amino acid metabolism, carbohydrate metabolism and energy metabolism (Supplementary Data 6). The small set of genes encoding these core protein families were also the dominant fraction of the metagenomes, accounting for more than 60% of reads over the first 3 years of life. A median of 201,911 protein-encoding genes were predicted in each subject’s metagenome (Q1 = 129,145 and Q3 = 282,546), of which a median of 129,328 putative protein sequences belonged to the core protein families defined across the study data (Q1 = 86,034 and Q3 = 174,161).

Each core protein family consisted of protein-encoding genes obtained from an average of 548 subject metagenomes with varied geographic locations (United States or Europe), genders (male or female) and delivery modes (Cesarean section or vaginal). For each core protein family, the median percentage of subjects in the United States was 29%, the median percentage of male subjects was 54%, and the median percentage of subjects delivered vaginally was 74% (Supplementary Fig 3). These values closely approximate the demographic metadata of the entire subject cohort, indicating that important subject variables (geographic location, gender and delivery mode) are well-represented in the core protein families.

Core protein families were clustered based on the longitudinal profiles of their average abundances over eight developmental stages from 3 to 35 months of age. The 64,142 core protein families were clustered into 10 clusters with distinct temporal patterns (Supplementary Fig 4). Divergent KEGG modules were found to be over-represented in these clusters (Supplementary Data 7). This reflected the companion functional changes along with the community composition changes (Fig. 2) during early childhood.

We then investigated the longitudinal changes of functional capacity in major orders of the microbiomes over the 8 developmental stages (Fig. 3e). The *Enterobacterales* and *Clostridiales* orders had shifting functional profiles over time as shown in the first two principal components of the KEGG categories. However, the other major orders showed stable functional profiles over time (*p*-value = 1.0, Multiple response permutation procedure). In contrast, there were significant longitudinal changes of taxonomic compositions in major functional categories of the microbiomes over time (*p*-value = 0.001, Multiple response permutation procedure) (Fig. 3f), reflecting major changes in community composition during early childhood.

### Association of IA seroconversion with microbiota dysbiosis

A subject was considered to have IA seroconversion if one of the three islet autoantibodies, MIAA, GAD or IA2A, was detected. We hypothesized that seroconversion of many subjects may be associated with the metagenomic abundance changes of certain microorganisms and functions (i.e., MAGs and protein families, respectively) in the gut microbiota. Generalized linear mixed models (GLMM)^26^ were used to identify core protein families that were significantly associated with the IA seroconversion status of fecal samples as a fixed effect. The TEDDY cohort contained 660 subjects who donated at least 4 samples and had IA seroconversion status information available. Of the 660 subjects, IA seroconversion was observed in 253 subjects, who donated 3,129 fecal samples before seroconversion (IA status = 0) and 1,504 fecal samples after seroconversion (IA status = 1). Seroconversion was not observed in 307 subjects before they exited the study and these subjects donated 5951 samples (IA status = 0). The subject-specific effects were controlled by including subject IDs as a random effect in the GLMM models. Sample collection age and subject HLA haplotype were included as fixed effects in the GLMM models. The effect of IA status and the effect of age at sample collection can be resolved in the GLMM models because the 5951 samples from the 307 subjects without seroconversion served as the baseline for IA status = 0 across all the developmental stages. Core protein families were tested for associations because these were present in more than half of the subjects, allowing for sufficient statistical power. GLMM identified 5346 core protein families with an estimated false discovery rate of 0.03, which showed significantly different gene abundances between the post-seroconversion samples and the control samples (Supplementary Data 8). Positive association with IA seroconversion was found in 2190 families, while 3156 families were negatively associated with IA seroconversion.

Similar to 16 S rRNA genes from a microbial clade, protein-coding genes from a core family were considered in this study as a proxy for the metagenomic abundances of the microorganisms harboring these genes. As would be expected, based on this approximation, there were no MAGs containing both positively IA-associated protein families and negatively IA-associated protein families (Supplementary Data 9). A total of 2373 MAGs were significantly enriched in positively IA-associated protein families and 1549 MAGs in negatively IA-associated protein families (Fisher’s exact test, one-sided, *q*-value <  0.01; Supplementary Data 9).

**Fig. 4:**
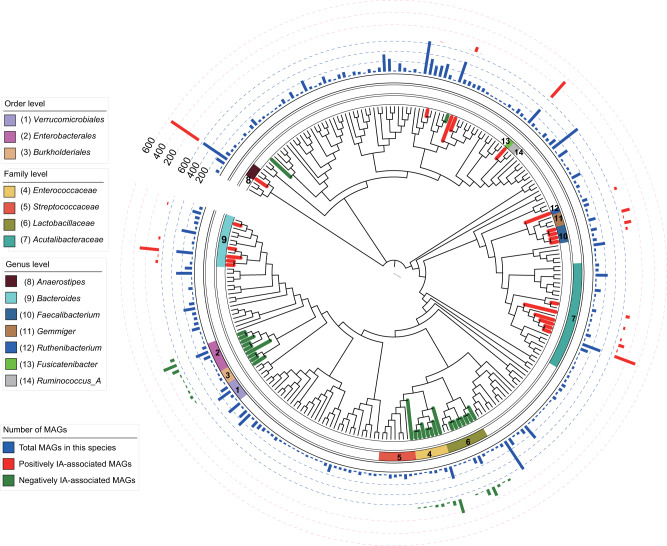
Taxonomy distribution of MAGs positively or negatively associated with seroconversion. For clarity, the phylogenetic tree comprises only species containing more than 10 high-quality MAGs. The inner ring shows the total numbers of MAGs in each species in blue bars and the outer ring shows the numbers of positively IA-associated MAGs in red bars and negatively IA-associated MAGs in green bars. Branches of the phylogenetic trees are colored in red for taxa containing positively IA-associated MAGs and in green for taxa containing negatively IA-associated MAGs. Taxa are highlighted in arcs of varying colors and are identified in the legend. Comparative genomics were performed between adjacent taxa with and without significant MAGs. Source data are provided as a Source Data file.

While this approach considered every MAG as a collection of proteins in core protein families, the distributions of the 2373 MAGs with positive IA-association and the 1549 MAGs with negative IA-association were highly clustered based on their taxonomy classification (Fig. 4, Supplementary Fig 5 and Supplementary Data 10). The positively IA-associated MAGs belonged to 41 species in the orders *Bacteroidales*, *Lachnospirales* and *Oscillospirales*. The negatively IA-associated MAGs primarily originated from 90 species in the orders *Lactobacillales*, *Burkholderiales* and *Enterobacterales*.

### Comparative genomics analysis of MAGs associated with IA seroconversion

We hypothesized that MAGs with positive or negative association with IA seroconversion are enriched in certain taxa-specific metabolic pathways that may play a role in triggering or delaying seroconversion. Comparative genomics analyses were conducted on five taxonomic clades that contained MAGs positively associated with IA seroconversion, including the *Acutalibacteraceae* family, the *Ruminococcaceae* family (including the *Faecalibacterium* genus and *Ruthenibacterium* genus), the *Bacteroides* genus, the *Anaerostipes* genus, and the *Fusicatenibacter* genus. The positive MAGs in these taxa accounted for 96.25% of all positive MAGs. Positive MAGs from these taxa were compared with MAGs with insignificant IA-association that were assigned to the same taxonomic ranks from adjacent lineages. Table 1 shows KEGG modules that were significantly over-represented in the MAGs with positive IA-association, according to both enrichment analysis and phylogenetic logistic regression. Similarly, comparative genomic analyses were conducted on two taxonomic clades that contained a large number of MAGs negatively associated with seroconversion, including the *Lactobacillaceae and Enterococcaceae* families in the *Lactobacillales* order and the combined *Burkholderiales* and *Enterobacterales* orders (Table 2). The negative MAGs in these taxa accounted for 98.97% of all negative MAGs. For both positive and negative MAGs, a separate GLMM analysis was conducted using MAG abundances to independently validate the statistical significance of these MAG groups’ association with seroconversion. The abundances of the MAGs in the five positive groups and the two negative groups were found to have significant association with IA seroconversion as a fixed effect, while those in the control groups did not (Supplementary Data 11).

**Table 1:** Comparative genomics of the taxa containing MAGs positively associated with IA seroconversion.

Major species containing positive MAGs (Positive MAGs/Total MAGs)	Module ID	Function description	Enrichment analysis^b^		Phylogenetic regression analysis^c^	
			Effect size	*Q value*	Estimate	*Q* value
Comparison within *g_Bacteroides (*^a^ #9*)*						
*Bacteroides uniformis (*312*/* 327*)*	M00064	ADP-L-glycero-D-manno-heptose biosynthesis	0.43	2.16E–32	1.95	6.74E–4
*Bacteroides cellulosilyticus (*41*/* 43*)*						
*Bacteroides intestinalis (*11*/*11*)*						
*Bacteroides stercoris (*31*/* 44*)*						
*Bacteroides ovatus (*18*/* 170*)*						
Comparison within *g__Anaerostipes (*^a^ #8*)*						
*Anaerostipes hadrus (*540*/* 565*)*	M00616	Sulfate-sulfur assimilation	1.00	3.05E–40	7.22	2.53E–26
	M00307	Pyruvate` oxidation, pyruvate => acetyl-CoA	0.97	4.52E–38	9.26	3.80E–13
	M00620	Incomplete reductive citrate cycle, acetyl-CoA => oxoglutarate	0.95	8.18E–39	24.47	5.60E–05
	M00173	Reductive citrate cycle (Arnon-Buchanan cycle)	0.79	3.69E–33	11.32	7.04E–15
	M00596	Dissimilatory sulfate reduction, sulfate => H2S	0.78	6.96E–30	12.67	4.67E–18
	M00176	Assimilatory sulfate reduction, sulfate => H2S	0.71	2.71E–23	17.31	2.24E–21
	M00374	Dicarboxylate-hydroxybutyrate cycle	0.62	9.19E–22	11.02	4.57E–12
	M00632	Galactose degradation, Leloir pathway, galactose => alpha-D-glucose-1P	0.58	8.72E–15	11.47	9.30E–16
	M00125	Riboflavin biosynthesis, GTP => riboflavin/FMN/FA	0.48	1.97E–14	7.84	1.03E–15
	M00376	3-Hydroxypropionate bi-cycle	0.47	4.68E–12	7.67	3.67E–17
	M00565	Trehalose biosynthesis, D-glucose 1P => trehalose	0.45	3.87E–12	10.52	6.69E–18
	M00017	Methionine biosynthesis, apartate => homoserine => methionine	0.45	2.50E–14	8.07	1.40E–14
	M00159	V-type ATPase, prokaryotes	0.43	2.37E–14	14.89	7.99E–23
	M00082	Fatty acid biosynthesis, initiation	0.43	1.23E–11	7.08	9.69E–16
	M00028	Ornithine biosynthesis, glutamate => ornithine	0.41	4.15E–10	12.05	2.11E–12
Comparison within *f__Acutalibacteraceae (*^a^ #7*)*						
*Ruminococcus_E bromii (*16*/*16*)*	M00616	Sulfate-sulfur assimilation	1.11	2.52E–159	12.24	4.48E–11
*Ruminococcus_E bromii_B (*357/372*)*	M00596	Dissimilatory sulfate reduction, sulfate => H2S	0.85	3.90E–112	9.18	2.17E–12
*Ruminococcus_E sp*003526955 *(*95/96*)*	M00176	Assimilatory sulfate reduction, sulfate => H2S	0.80	3.79E–112	8.84	6.1793E–13
*Ruminococcus_H sp003531055 (64/78)*						
*GCA-900066995 sp*900291955 *(33/33)*						
*Ruminococcus_E sp*900314705 *(*24/25*)*						
Comparison of *g__Fusicatenibacter (*^a^ #13)* versus g__Ruminococcus_A*(^a^ #14)						
*Fusicatenibacter saccharivorans*(335/346)	M00019	Valine/isoleucine biosynthesis, pyruvate => valine / 2-oxobutanoate => isoleucine	0.65	2.31E–17	9.50	1.50E–06
	M00570	Isoleucine biosynthesis, threonine => 2-oxobutanoate => isoleucine	0.62	1.20E–14	9.11	1.37E–06
	M00432	Leucine biosynthesis, 2-oxoisovalerate => 2-oxoisocaproate	0.57	1.48E–14	9.97	2.93E–05
	M00535	Isoleucine biosynthesis, pyruvate => 2-oxobutanoate	0.57	2.06E–12	28.20	1.09E–05
	M00115	NAbiosynthesis, aspartate => NA	0.56	1.55E–12	2.05	8.11E–05
	M00346	Formaldehyde assimilation, serine pathway	0.52	3.52E–10	4.69	3.79E–04
	M00017	Methionine biosynthesis, apartate => homoserine => methionine	0.46	8.56E–08	30.33	6.47E–13
	M00007	Pentose phosphate pathway, non-oxidative phase, fructose 6P => ribose 5P	0.45	1.12E–08	6.96	2.05E–07
Comparison of *g__Faecalibacterium (*^a^ #10*) and g__Ruthenibacterium (*^a^ #12*) versus g__Gemmiger (*^a^ #11*)*						
*Faecalibacterium prausnitzii_C (*34*/*36*)*	M00651	Vancomycin resistance, D-Ala-D-Lac type	0.45	6.08E–12	3.49	1.69E–07
*Faecalibacterium prausnitzii_D*(134/137)	M00173	Reductive citrate cycle (Arnon-Buchanan cycle)	0.42	2.40E–11	7.69	1.36E–07
*Faecalibacterium prausnitzii_G (*111*/*114*)*						
*Faecalibacterium sp900539885 (*12*/*12*)*						
*Ruthenibacterium lactatiformans (*42/99)						

^a^The numbers correspond to the numbered taxa shown in the caption of Fig. 4.

^b^Wilcoxon test (two-sided), Benjamini-Hochberg adjusted

^c^Phylogenetic linear modeling (two-sided), Benjamini-Hochberg adjusted

**Table 2:** Comparative genomics of the taxa containing MAGs negatively associated with IA seroconversion.

Major species containing negative MAGs (Negative MAGs/Total MAGs)	Module ID	Function description	Enrichment analysis^b^		Phylogenetic regression analysis^c^	
			Effect size	*Q* value	Estimate	*Q* value
Comparison of *f__Enterococcaceae (*^a^ #4) and *f__Lactobacillaceae (*^a^ #6) versus *f__Streptococcaceae (*^a^ #5)						
*Enterococcus faecalis*(228/245)	M00550	Ascorbate degradation, ascorbate => D-xylulose−5P	1.07	3.78E–125	3.92	1.97E–10
*Enterococcus_A avium*(76/79)	M00061	D-Glucuronate degradation, D-glucuronate => pyruvate + D-glyceraldehyde 3P	0.79	4.94E–89	4.95	8.80E–18
*Enterococcus_B faecium*(51/55)	M00631	D-Galacturonate degradation (bacteria), D-galacturonate => pyruvate + D-glyceraldehyde 3P	0.76	1.40E–83	6.43	2.15E–18
*Enterococcus_B faecium_B*(18/20)	M00008	Entner-Doudoroff pathway, glucose-6P => glyceraldehyde-3P + pyruvate	0.73	1.48E–76	5.45	1.25E-12
*Enterococcus_D casseliflavus* (19/21)	M00006	Pentose phosphate pathway, oxidative phase, glucose 6P => ribulose 5P	0.68	1.85E–67	8.24	4.79E–28
*Enterococcus_D gallinarum* (40/42)	M00003	Gluconeogenesis, oxaloacetate => fructose-6P	0.65	1.68E–75	6.53	1.12E–15
*Enterococcus_D sp002850555* (12/12)	M00116	Menaquinone biosynthesis, chorismate => menaquinol	0.61	4.76E−50	10.07	2.55E–10
*Lacticaseibacillus paracasei*(100/111)	M00153	Cytochrome bd ubiquinol oxidase	0.60	3.42E–59	9.35	1.31E–05
*Lacticaseibacillus rhamnosus*(153/165)	M00308	Semi-phosphorylative Entner-Doudoroff pathway, gluconate => glycerate-3eP	0.54	9.00E–48	6.59	3.99E–21
*Lactiplantibacillus plantarum (33/33)*	M00004	Pentose phosphate pathway (Pentose phosphate cycle)	0.53	2.15E–51	14.10	1.88E–28
*Lactobacillus gasseri* (19/21)	M00165	Reductive pentose phosphate cycle (Calvin cycle)	0.52	1.99E–47	7.70	3.94E–18
*Lactobacillus paragasseri* (8/13)	M00011	Citrate cycle, second carbon oxidation, 2-oxoglutarate => oxaloacetate	0.47	7.77E–32	7.83	7.86E–28
*Limosilactobacillus fermentum (*44/49)	M00532	Photorespiration	0.46	1.02E–36	6.28	6.63E–39
*`Limosilactobacillus oris (1*6*/16)*	M00001	Glycolysis (Embden-Meyerhof pathway), glucose => pyruvate	0.46	1.63E–43	19.39	7.62E–26
*Lactococcus lactis (*16/28)	M00167	Reductive pentose phosphate cycle, glyceraldehyde-3P => ribulose-5P	0.43	6.08E–28	13.87	1.93E–19
	M00345	Formaldehyde assimilation, ribulose monophosphate pathway	0.43	5.47E–31	19.86	6.85E–10
Comparison of *o__Burkholderiales (*^a^ #3) and *o__Enterobacterales (*^a^ #2) versus *o__Verrucomicrobiales (*^a^ #1)						
*Parasutterella excrementihominis*(45/45)	M00529	Denitrification, nitrate => nitrogen	1.20	1.15E–137	20.59	9.55E–08
*Parasutterella sp000980495* (26/27)	M00880	Molybdenum cofactor biosynthesis, GTP => molybdenum cofactor	1.08	3.82E-128	11.37	2.60E-17
*Sutterella wadsworthensis (*14/14)	M00550	Ascorbate degradation, ascorbate => D-xylulose-5P	0.96	3.08E–105	3.87	5.24E–09
*Enterobacter himalayensis* (15/18)	M00804	Complete nitrification, comammox, ammonia => nitrite => nitrate	0.81	8.55E-83	8.19	4.38E-29
*Escherichia coli* (226/255)	M00150	Fumarate reductase, prokaryotes	0.78	2.43E–88	15.61	9.55E–09
*Escherichia coli_D* (69/76)	M00616	Sulfate-sulfur assimilation	0.69	1.12E–72	4.31	1.12E–22
*Escherichia flexneri* (114/130)	M00095	C5 isoprenoid biosynthesis, mevalonate pathway	0.69	5.94E–71	5.76	2.92E–16
*Klebsiella_A oxytoca* (12/13)	M00718	Multidrug resistance, efflux pump MexAB-OprM	0.67	5.63E–61	21.27	3.85E–43
*Klebsiella pneumoniae* (19/25)	M00546	Purine degradation, xanthine => urea	0.65	8.20E–58	4.92	1.44E–19
	M00167	Reductive pentose phosphate cycle, glyceraldehyde-3P => ribulose-5P	0.64	2.99E–56	16.59	1.08E–44
	M00879	Arginine succinyltransferase pathway, arginine => glutamate	0.63	3.97E–64	3.93	4.92E–21
	M00087	beta-Oxidation	0.62	1.39E–61	3.42	9.28E–09
	M00761	Undecaprenylphosphate alpha-L-Ara4N biosynthesis, UDP-GlcA => undecaprenyl phosphate alpha-L-Ara4N	0.56	5.70E–51	2.89	4.27E–08
	M00417	Cytochrome o ubiquinol oxidase	0.55	3.85E–51	2.89	2.06E–08
	M00170	C4-dicarboxylic acid cycle, phosphoenolpyruvate carboxykinase type	0.53	5.49E–42	15.96	9.14E–50
	M00004	Pentose phosphate pathway (Pentose phosphate cycle)	0.52	2.09E–40	22.59	1.10E–17
	M00088	Ketone body biosynthesis, acetyl-CoA => acetoacetate/3-hydroxybutyrate/acetone	0.51	6.75E–43	17.61	1.11E–11
	M00006	Pentose phosphate pathway, oxidative phase, glucose 6P => ribulose 5P	0.50	7.30E–40	5.24	5.18E–16
	M00615	Nitrate assimilation	0.49	4.46E–38	13.86	2.17E–37
	M00008	Entner-Doudoroff pathway, glucose-6P => glyceraldehyde-3P + pyruvate	0.48	7.42E–39	9.68	7.37E–08
	M00165	Reductive pentose phosphate cycle (Calvin cycle)	0.48	1.03E–38	18.87	7.92E–22
	M00061	D-Glucuronate degradation, D-glucuronate => pyruvate + D-glyceraldehyde 3P	0.47	2.22E–40	2.89	8.20E–15
	M00345	Formaldehyde assimilation, ribulose monophosphate pathway	0.45	3.02E–30	19.62	3.65E–03
	M00034	Methionine salvage pathway	0.43	4.56E–27	18.65	3.89E–38
	M00579	Phosphate acetyltransferase-acetate kinase pathway, acetyl-CoA => acetate	0.42	2.03E–30	4.47	1.24E–13
	M00631	D-Galacturonate degradation (bacteria), D-galacturonate => pyruvate + D-glyceraldehyde 3P	0.41	6.69E–31	4.41	9.77E–26

^a^The numbers correspond to the numbered taxa shown in the caption of Fig. 4.

^b^Wilcoxon test (two-sided), Benjamini-Hochberg adjusted

^c^Phylogenetic linear modeling (two-sided), Benjamini-Hochberg adjusted

The *Bacteroides* genus contained 1196 MAGs, of which none were negatively associated with IA seroconversion and 422 were positively associated with seroconversion. 413 of the 422 positive MAGs originated from five species: *Bacteroides uniformis, Bacteroides cellulosilyticus, Bacteroides intestinalis, Bacteroides stercoris* and *Bacteroides ovatus*. Positive IA-association was found with 312 out of 327 MAGs in *Bacteroides uniformis*, 41 out of 43 MAGs in *Bacteroides cellulosilyticus*, all 11 MAGs in *Bacteroides intestinalis*, 31 out of 44 MAGs in *Bacteroides stercoris* and 18 out of 170 MAGs in *Bacteroides ovatus*. Elevated abundances of the *Bacteroides* genus, including *Bacteroides uniformis and Bacteroides ovatus* at the species level, have been reported in seroconverted and T1D subjects^8,27,28^ Findings of elevated abundance of *Bacteroides cellulosilyticus*, *Bacteroides intestinalis* and *Bacteroides stercoris* have been reported in T1D patients^29^ Our analysis of MAGs confirmed the positive and consistent signal of IA-association with *Bacteroides uniformis, Bacteroides cellulosilyticus*, *Bacteroides intestinalis*, *Bacteroides stercoris*, and *Bacteroides ovatus*.

In the *Bacteroides* genus, genes involved in the ADP-L-glycero-D-manno-heptose biosynthesis pathway, a key step in the biosynthesis of lipopolysaccharides (LPS), were enriched in 422 positively IA-associated MAGs compared with 774 MAGs lacking significant association with IA seroconversion. Moreover, the other three module pathways of LPS biosynthesis, including M00060 (KDO2-lipid A biosynthesis, LpxL-LpxM type), M00063 (CMP-KDO biosynthesis) and M00866 (KDO2-lipid A biosynthesis, non-LpxL-LpxM type), were positively associated with IA seroconversion using phylogenetic logistic regression, although not detected using enrichment analysis (Supplementary Data 12). LPS is a major component of the outer membranes of Gram-negative bacterial species, and many studies implicate bacterial LPS in the modulation of the host immune system in ways that influence the onset of T1D^8,30,31^ and T2D^32,33^ In particular, Vatanen, et al.^30^ showed that the LPS produced by the *Bacteroides* species in gut microbiota has immunoinhibitory properties that may impede early immune education and contribute to the development of T1D. Our finding of a positive IA-association with genes of *Bacteroides* LPS biosynthesis not only supports these previous findings, but also validates our comparative genomics approach for targeting key MAGs and functions related to IA seroconversion in a taxa-specific manner.

Dissimilatory sulfate reduction, assimilatory sulfate reduction, and sulfate-sulfur assimilation pathways were enriched in MAGs with positive IA-association from both the *Anaerostipes* genus and the *Acutalibacteraceae* family (Table 1). These positive MAGs were concentrated in strains of *Anaerostipes hadrus* and several unclassified species related to *Ruminococcus bromii*. These metabolic pathways lead to the reduction of sulfate to form either hydrogen sulfide (H_2_S) through anaerobic respiration or the sulfur-containing amino acids cysteine and methionine through assimilation into biomass. Previous studies have shown that high concentrations of H_2_S from sulfate-reducing bacteria in gut microbiota can adversely affect the bowel environment by increasing toxicity and lowering pH, contributing to the immune response and to inflammatory activation in the gut^34–37^ Excessive H_2_S may break the crosslinking disulfide bonds in intestinal mucins, leading to decreased mucus viscosity and increased permeability across the mucus layer^38,39^ We hypothesize that elevated abundance of sulfate-reducing bacteria from *Anaerostipes hadrus* may contribute to gut barrier disruption and immune response activation in the gut, promoting IA seroconversion.

MAGs that were negatively associated with seroconversion were concentrated in a few taxa, including the *Enterococcaceae* and *Lactobacillaceae* families in the *Lactobacillales* order and two *Proteobacteria* orders, *Burkholderiales* and *Enterobacterales*. The metabolic pathways enriched in these negatively IA-associated MAGs were identified in two comparative analyses (Table 2). Negatively IA-associated MAGs in the family *Enterococcaceae* were mostly from *Enterococcus* or related genera, including 228 in *Enterococcus faecalis*, 76 in *Enterococcus_A avium*, 51 in *Enterococcus_B faecium*, and 40 in *Enterococcus_D gallinarum*. Negatively IA-associated MAGs in the family *Lactobacillaceae* were mostly from *Lacticaseibacillus* genera, including 100 in *Lacticaseibacillus paracasei*, 153 in *Lacticaseibacillus rhamnosus* and 33 in *Lactiplantibacillus plantarum*. Notably, *Lacticaseibacillus rhamnosus* was previously found to have higher abundance in control subjects in comparison with IA-seroconverted subjects^6^ Many pathways enriched in these negatively IA-associated MAGs were involved in carbohydrate degradation (Table 2). These *Enterococcus* and *Lactobacillaceae* species are lactic acid bacteria with probiotic properties^40–42^ When supplemented in diets of both mice and humans, *Enterococcus faecalis* and *Enterococcus_A avium* increased SCFA production via modulation of the gut microbiome^40^ *Lactobacillus plantarum* (*Lactiplantibacillus plantarum* subsp. plantarum) restored the impaired mucus barrier of the proximal colon in a mouse model of accelerated aging^43^ These findings suggest IA seroconversion may be delayed or prevented by these known probiotic microorganisms.

The *Enterobacterales* order contained 410 MAGs from the *Enterobacteriaceae* family negatively associated with seroconversion, including 296 MAGs in *Escherichia coli* and 114 MAGs in *Escherichia flexneri*. The *Burkholderiales* order contained 76 negative MAGs from the *Parasutterella* species. *Escherichia coli* LPS was shown to induce protective endotoxin tolerance and delay T1D onset in the non-obese diabetic (NOD) mouse model of spontaneous development of T1D^30^ In a longitudinal microbiome study, Tetz, et al.^44^ showed that *Escherichia coli* was depleted prior to seroconversion. Findings of diminished abundance of *Parasutterella* species has been described in children with T1D^29^ The negatively IA-associated MAGs in these two orders were compared with MAGs from the adjacent *Akkermansia* order which were not associated with seroconversion (Fig. 4 and Supplementary Fig 5). Our comparative genomics analysis identified denitrification as the most enriched pathway in these MAGs. As dietary nitrate intake is a risk factor for T1D^45–47^, we hypothesize that these microorganisms may offer protective effects against T1D by reducing the nitrate level in the gut through denitrification.

## Discussion

Due to the strong variability in gut microbiome composition persistently observed across individuals^48,49^, a central objective in human gut microbiome research has been to define a ‘core’ microbiome at a population scale. While the idiosyncratic components of an individual’s microbiome may causally contribute to his/her phenotype, only the findings from core microbiomes can be generalized across many individuals and can form the basis for future dietary or therapeutic interventions in general populations. Many studies have used bacterial lineages as the units to define core gut microbiomes, based on 16 S rRNA amplicon sequencing or metagenomics sequencing results of large cohorts^50–54^ However, even within a bacterial species, there is substantial strain variability represented by core genomes and pan-genomes in comparative genomics studies^55,56^ Ideally, one may define core microbiomes as the core genomes of core bacterial lineages. Our median mapping rate to the metagenome assemblies was 89%, indicating that most of the genes encoded in the TEDDY gut microbiomes can be captured by the metagenome assemblies. Thus, we tested the hypothesis that there was a core microbiome defined by a set of highly prevalent protein families that were shared across the majority of subjects (>50%) in the TEDDY cohort. We found there were 64,142 protein families that met this standard and that were present without significant representational biases in terms of developmental stages, genders, geographic locations and delivery modes. Based on the read mapping rates for an average subject in this cohort, 64% of their sequenced microbiome DNA can be attributed to genes in these core protein families, 25% of their microbiome DNA to genes in peripheral protein families, and 11% to unassembled genes, supporting our hypothesis of a large core microbiome and a small peripheral microbiome in this cohort. This represented an alternative approach to defining core microbiomes in large-scale metagenomic sequencing datasets.

A total of 21,536 high-quality MAGs were recovered from 887 subjects, including high-quality MAGs for 176 previously undescribed species. This further expanded the diversity of the human gut MAG collection, especially for early childhood microbiomes. While efforts by Almeida, et al.^21^, Nayfach, et al.^18^ and Pasolli, et al.^22^ combined a large number of mostly cross-sectional studies, our results showed the advantage of subject-specific metagenome co-assembly and temporal co-variance binning in a long-term, multi-center, longitudinal metagenomics study. This approach achieved high mapping rates of metagenomic reads, including 89% on metagenome assemblies, 70% on median- or high-quality MAGs, and 51% on high-quality MAGs. It is important for a metagenomics analysis to obtain a high mapping rate, because the reads not accounted for by the metagenomics analysis represent genes and organisms that may be omitted in subsequent investigations. The streetlight effect^57^ of a metagenomics analysis results in missed discoveries in the unobserved parts of microbiomes and increased variabilities when comparing the observed parts of microbiomes. Here, we demonstrated that high mapping rates can be achieved to reduce the streetlight effect in a long-term, multi-center, longitudinal metagenomics study using subject-specific metagenome co-assembly and temporal co-variance binning.

To identify microorganisms associated with IA seroconversion, subject-specific metagenomes were compared based on a core microbiome defined by protein families. The metagenomic abundances of a protein family in different microbiomes are proxies of the relative abundances of their originating microorganisms that may or may not have corresponding MAGs. Thus, a significant core protein family can be considered to represent a single-gene-based grouping of microorganisms that have different abundances in the fecal metagenomes in association with IA seroconversion. A total of 2373 MAGs were significantly enriched in the 2190 protein families with positive IA-association and 1,549 MAGs were enriched in the 3156 protein families with negative IA-association. The IA-association of these MAGs grouped by their taxonomy were confirmed by the abundance changes of the MAGs themselves. While previous association studies implicated specific taxa or functions in T1D pathogenesis or IA seroconversion, our study implicated specific MAGs. In comparison with taxa- or function-defined associations in previous studies, an advantage of MAG-defined association is that it enables comparative genomics analyses that control for broad evolutionary differences between taxa and specifically identify the functional biases associated with IA for a clade of phylogenetically-related organisms. Here, these analyses allowed for detection of functions specific to a given set of IA-associated strains, as compared with a closely related sister clade without IA association. This approach was validated by our re-discovery of the association of *Bacteroides* LPS biosynthesis with IA seroconversion in the TEDDY cohort^8,30,31^ This approach was then used to generate hypotheses regarding IA seroconversion, including the potential detrimental effects of *Anaerostipes* sulfate reduction, and the potential protective effects of lactic acid bacteria and *Escherichia* nitrate reduction.

By its nature, our analysis only suggests association, rather than causation, whereby the IA-associated MAGs could be the cause or the effect of IA seroconversion of the subject. As shown in Vatanen, et al.^30^ and Han, et al.^31^, the value of an association analysis is to provide specific hypotheses that can be tested in mechanistic experiments. An advantage of MAG-defined associations over taxa- or function-defined associations is to enable a more precise selection of microbial strains for validation experiments in animal models. Because of the large strain heterogeneity in many microbial species^58,59^, we postulate that future validation studies will be more likely to succeed using strains whose genomes closely match MAGs with disease association. Eventually, this may lead to strain-level precision intervention strategies against Type-1 diabetes by promoting or suppressing specific microbial strains in personal microbiota using probiotics and/or prebiotics during critical windows of disease progression.

## Methods

### Retrieval of TEDDY data

All metagenome sequencing data and clinical data were obtained from The Environmental Determinants of Diabetes in the Young (TEDDY) Study, a longitudinal study of subjects with either a genetic predisposition for T1D or at least a first-degree relative with T1D. A total of 13,245 metagenome sequencing runs from a time-course collection of 12,276 fecal samples from 887 subjects were downloaded from NCBI dbGap using SRA Toolkit tools v2.9.6. The fecal samples were collected approximately monthly from 3 to 48 months of age, thereafter every three months until 72 months of age. Collections were carried out by six clinical centers in four countries (Finland, Germany, Sweden, and the United States). The clinical data were obtained from the NIDDK Central Repository at https://repository.niddk.nih.gov/studies/teddy/.

### Metagenome assembly and abundance-series binning

The raw reads of all samples from each subject were co-assembled using SPAdes v3.13.1 in the metaSPAdes mode^60^ Scaffolds longer than 2 kb were used to bin the metagenome-assembled genomes (MAGs) using MetaBAT 2 v2.12.1^61^ with default parameters. Binning was based on the abundance co-variation of an organism’s scaffolds across all samples from a subject. Pullseq v1.0.2 was used to filter scaffolds by a minimum length. Sequencing reads in the individual samples were mapped onto their corresponding metagenome with Bowtie 2 v2.3.5.1. After removal of the unmapped reads using shrinksam v0.9.0, coverage depths of scaffolds were calculated using samtools v0.1.19 (‘samtools view -Sbu’ followed by ‘samtools sort’) and the jgi_summarize_bam_contig_depths function from MetaBAT 2 v2.12.1. The quality of MAGs was estimated using CheckM v1.1.2 with lineage_wf workflow. Based on the criteria established in the minimum information about a metagenome-assembled genome (MIMAG) standard^23^, the MAGs obtained were classified into high-quality MAGs (completeness > 90% and contamination <5%) and medium-quality MAGs (completeness > = 50% and contamination <10%). The Reads Per Kilobase per Million reads (RPKM) of a MAG in every sample was calculated based on the total number of reads mapped onto its scaffolds and the total length of its scaffolds. All the high-quality MAGs have been deposited in the European Nucleotide Archive (ENA) under accession PRJEB40730 (https://www.ebi.ac.uk/ena/browser/view/PRJEB40730).

### Taxonomy assignment and clustering of MAGs

Taxonomy classifications of high-quality MAGs were inferred using GTDB-Tk v1.3.0 based on reference species in the Genome Taxonomy Database (https://gtdb.ecogenomic.org/; GTDB Release 95)^62^ If multiple MAGs were assigned to a GTDB species, the MAG with the highest quality score, defined as completeness – (5 × contamination)^63^, was selected as the representative MAG for the species. If multiple MAGs had the same highest quality score, the MAG with the largest genome length was selected. MAGs assigned to the genus level or above were clustered into metagenomic species based on 95% average nucleotide identity (ANI) using dRep v2.4.0 (gANI -pa 0.9 -sa 0.95 -nc 0.6). A representative MAG was selected for each cluster based on quality scores and genome size as described above. In total, this procedure generated 883 species from 21,536 high-quality MAGs in the TEDDY dataset. A phylogenetic tree of the 883 species were inferred based on their representative MAGs using PhyloPhlAn 2.0^64^ The phylogenetic tree was plotted and annotated using iTOL v5 (https://itol.embl.de/).

### Identification of new species in the human gut microbiome

MAGs recovered in this study were compared with MAGs obtained from three previous large-scale surveys of the human gut microbiome [Almeida, et al.^21^, Nayfach, et al.^18^ and Pasolli, et al.^22^] and used as reference databases. Almeida, et al.^21^ recovered 39,891 high-quality MAGs from 13,133 human gut microbiome datasets from 75 different studies. Nayfach, et al.^18^ recovered 24,345 high-quality MAGs from 3810 globally distributed, diverse human subjects and clustered these MAGs, along with the reference genomes from PATRIC and IMG, into 23,790 species, which included 2935 human gut species with a high-quality genome^18^ Pasolli, et al.^22^ recovered 154,723 microbial genomes (70,178 high quality) from 9428 metagenomes spanning body sites, ages, countries, and lifestyles. A new species was defined as a species not detected in any of the reference genomes at a threshold of ANI > 95%. The function ‘mash sketch’ from Mash version 2.2 was used to convert the reference genomes into a MinHash sketch with default k-mer and sketch sizes. Then, the Mash distance between each MAG and the set of reference genomes was calculated with ‘mash dist’ to find the best match (i.e., the reference genome with the lowest Mash distance) requiring distance < 0.2, corresponding to identity > 80%. Subsequently, each MAG and its closest relative were aligned using an ANI calculation tool, ANIcalculator v1.0, to compare each pair of genomes, reporting the fraction of the MAG that was aligned (aligned query, AQ) and ANI.

### Time-course clustering of species

The abundance of a species within a developmental stage was estimated as the average RPKM of all the MAGs from the species that were obtained from all fecal samples collected during the developmental stage. Eight developmental stages were considered, including 3–5 months of age, 6–8 months of age, 9–11 months of age, 12–15 months of age, 16–19 months of age, 20–23 months of age, 24–29 months of age, and 30–35 months of age. Species were clustered to seven clusters based on their abundances across the 8 developmental stages using the fuzzy C-means clustering algorithm in the R library ‘Mfuzz’ version 2.54.0^65,66^ The number of clusters was selected using the elbow method based on the Ball-Hall index^67^, which was calculated using the R package clusterCrit (version 1.2.8)^68^ Species with missing abundance values in more than 25% of the time points were filtered.

### Construction, function annotation, and time-series clustering of core protein families

Using Prodigal v2.6.3 (option –p meta)^69^, proteins were predicted from all scaffolds >1 kbps that were assembled from subjects with at least four metagenome samples. A total of 158,247,178 proteins were generated. The minimum length cutoff was 50 amino acids. These proteins were clustered using a hierarchical clustering procedure for metagenomic sequence analysis as described in Li, et al.^70^ Briefly, proteins were clustered first at 90% identity, then at 65% identify, and finally at 40% identity using CD-HIT v4.8.1. In each step, proteins were divided into full-length open read frames (ORFs) with both start and stop codons, as well as fragmented ORFs with missing start and/or stop codons. Full-length ORFs were clustered with the following alignment coverage requirement on both long and short sequences: cd-hit -n 5 -d 0 -g 1 -p 1 -T 35 -M 0 -G 0 -aS 0.9 -aL 0.9. The fragmented ORFs were then recruited with the following partial alignment coverage requirement: cd-hit -n 5 -d 0 -g 1 -p 1 -T 35 -M 0 -G 0 -aS 0.9. Subsequently, cluster results from full-length ORFs and fragmented ORFs in each identity level were merged using the following parameters with -c to define the identity level: cd-hit-2d -n 5 -d 0 -g 1 -p 1 -T 35 -M 0 -G 0 -aS 0.9. This procedure generated a total of 288,586 protein families.

Protein functions were annotated using KO terms and MetaCyc reactions^71^ KO terms were assigned with KofamScan v1.0.0^72^ using default parameters. The top-ranked KO terms with scores above default thresholds were selected. Metacyc reactions were assigned to proteins based on homology searches against MetaCyc reference proteins in the MetaCyc database (https://metacyc.org/)^73^ The homology searches were conducted using DIAMOND (v0.9.26.127)^74^ with default parameters, with the exception of setting e-value < 0.0001 to use the sensitive mode. MetaCyc reactions of top-ranked reference proteins were included in the protein’s annotation information.

The gene abundance of a core protein family within a developmental stage was estimated as the average RPKM of all the protein-coding genes from this family in all fecal samples collected from this developmental stage. The core protein families were clustered to 10 clusters using the fuzzy C-means clustering algorithm in the R library ‘Mfuzz’ v2.54.0^65,66^ The number of clusters was selected using the elbow method based on the Ball-Hall index^67^, which was calculated using the R package clusterCrit (version 1.2.8)^68^ The KEGG modules enriched in each cluster of protein core families were identified by clusterProfiler (version 4.0.5, adjusted *p*-value < 0.05), using all the core protein families as the background annotation. The gene abundance of a KEGG functional category from an order was computed as the sum of the gene abundance of all the core protein families from this functional category in this order. Principal component analysis (PCA) analysis and Multiple Response Permutation Procedure (MRPP) were carried out using the ‘vegan’ package (version 2.5–7)^75^ PCA was performed on the functional category dimension in Fig. 3e and on the taxonomy dimension in Fig. 3f.

### Statistics & reproducibility

Generalized linear mixed modeling (GLMM)^26^ was used to test the statistical association between IA seroconversion and gene abundance changes within protein families. The read count, *K*_*ij*_, for protein family *i* in fecal sample *j* from subject *h* was expressed as a GLMM of the negative binomial family with a logarithmic link function^76^:

1 $${K}_{{ij}} \sim {{{{{\rm{NegativeBinomial}}}}}}({{{{{\rm{mean}}}}}}={\mu }_{{ij}},{{{{{\rm{dispersion}}}}}}={\delta }_{i})$$

2 $${{\log }}({\mu }_{{ij}})={\sum }_{k=1}^{6}{b}_{{ik}}{x}_{{jk}}+{r}_{{ih}}+{{\log }}\left({s}_{{ij}}\right)$$

3 $${r}_{{ih}} \sim {{{{{\rm{Normal}}}}}}({{{{{\rm{mean}}}}}}=0,{{{{{\rm{variance}}}}}}={\sigma }_{i}^{2})$$

4 $${s}_{{ij}}=\frac{{t}_{{ij}}}{\mathop{{mean}}\limits_{j}{(}{t}_{{ij}})}$$

*x*_*jk*_ and *b*_*ik*_ represented the seven fixed effects and their coefficients, respectively. The seven fixed effects were: [1] age at collection (an integer number of months), [2] delivery method (a categorical variable of caesarian or vaginal), [3] collection center (a categorical variable of SWE, FIN, GER, WAS, COL and GEO), [4] breastfeeding status (a categorical variable of yes or no), [5] solid food status (a categorical variable of yes or no), [6] HLA category (a categorical variable of DR4*030X/0302*DR3*0501/0201, DR4*030X/0302*DR8*0401/0402, DR4*030X/0302*DR1*0101/0501, DR4*030X/0302*DR4*030X/0302, DR3*0501/0201*DR3*0501/0201, DR4*030X/0302*DR13*0102/0604, Not*Eligible, DR4*030X/0302*DR9*030X/0303, DR3*0501/0201*DR9*030X/0303) and [7] seroconversion status (a categorical variable of yes or no). Breastfeeding status, solid food status and IA seroconversion status were coded based on the actual states of the subjects at the time of sample collection and, therefore, were varying during the time course of a subject. *r*_*ih*_ represented a random effect for subject *h*. *s*_*ij*_ is a sequencing-depth normalization factor for protein family *i* in sample *j* and it is calculated as the total number of reads of sample *j*, divided by the average total number of reads across all samples that are present in protein family *i*.

The R package ‘glmmTMB’ Version 0.2.3^77^ was used for fitting a GLMM model of the negative binomial family (family = nbinom2) for all fecal samples from each protein family. The *p*-values of the seroconversion fixed effect were extracted from the GLMM model fitting results. The *p*-values were adjusted using the Benjamini-Hochberg method^78^ for multi-comparison correction across all protein families.

Permutation testing^79^ was used to estimate an empirical false discovery rate for the fixed effect of IA seroconversion as follows: First, a decoy time series of the seroconversion status for every subject was generated using a random shuffling procedure. Samples collected before seroconversion were marked with 0 s and samples collected after seroconversion were marked with 1 s. Random shuffling was conducted by swapping the seroconversion time series of two semi-randomly selected subjects. The two subjects were required to have similar total numbers of samples in the time series and the number of seroconverted samples in either subject was required to be larger than the total number of samples in the other sample. These two requirements were designed to ensure that the swapping could simply exchange the 1 s in the back of the two time-series and pad 0 s in the front to maintain the lengths of the two time-series. The random shuffling did not alter the total number of samples before and after seroconversion in the cohort but changed the time of seroconversion for every subject. Many subjects were changed from having no seroconverted samples to having some seroconverted samples, or vice versa.

Next, the GLMM models were used to estimate the adjusted *p*-values of the seroconversion fixed effect for all protein families. The randomly shuffled time series of seroconversion status was used, but all other input data were not changed. All protein families in the permutation datasets which met the same filtering criteria as in the original dataset were considered to be false positives. The false discovery rate was calculated as the ratio of the number of false positive families identified in the permutation dataset to the number of positive families identified in the original dataset. This process was repeated ten times to estimate an average false discovery rate of the selected filtering criteria. Here, an adjusted *p*-value less than 10^−^^5^ and an effect size estimate larger than log_2_(1.3) were set as the filtering criteria, resulting in an estimated false discovery rate of 0.03.

Statistical tests, data analysis, and data visualization were conducted in R v3.6.3, Python v2.7.15, and Python v3.6.3. No statistical method was used to pre-determine the sample size. The experiments were not randomized and the allocation of subjects was not blind. Subjects with no clinical information on their IA seroconversion status or who donated less than 4 fecal samples were excluded from the analyses.

### KEGG module enrichment analysis for comparative genomics

An enrichment analysis was conducted to identify KEGG modules enriched in MAGs significantly associated with IA seroconversion, relative to reference MAGs in closely related lineages. Protein-coding sequences within all MAGs were annotated with KEGG KO terms. The protein frequency of a KEGG module in a MAG was calculated by counting the proteins annotated with KO terms belonging to the KEGG module. KEGG modules from plants, animals, fungi, and archaea were disregarded. Differential abundance analysis of protein frequencies in each KEGG module was conducted using the compositional data analysis tool ALDEx2 v1.18.0^80^ *P*-values were corrected to *q*-values using multiple testing with the Benjamini-Hochberg method. Phylogenetic logistic regression^81,82^ was performed using R package ‘phylolm’ (version 2.6)^83^ The dependent variable was a binary variable for the IA status (1 for IA-associated MAGs and 0 for non-associated). The gene counts in each KEGG module were defined as the independent variable. Significant modules that passed the filtering by both enrichment analysis (*q*-value < 10^−^^2^ and effect size > 0.4) and phylogenetic logistic regression (*q*-value < 10^−^^2^) are listed in Tables 1 and 2.

### Reporting summary

Further information on research design is available in the Nature Research Reporting Summary linked to this article.

## Supplementary information

Supplementary Information

Description of Additional Supplementary Files

Supplementary Data 1

Supplementary Data 2

Supplementary Data 3

Supplementary Data 4

Supplementary Data 5

Supplementary Data 6

Supplementary Data 7

Supplementary Data 8

Supplementary Data 9

Supplementary Data 10

Supplementary Data 11

Supplementary Data 12

Reporting Summary

## Data Availability

All high-quality MAGs generated in this study have been deposited in the European Nucleotide Archive (ENA) at EMBL-EBI under accession code PRJEB40730. The raw metagenomic sequencing data are available in the NCBI database of Genotypes and Phenotypes (dbGaP) under accession phs001442.v3.p2 (https://www.ncbi.nlm.nih.gov/projects/gap/cgi-bin/study.cgi?study_id=phs001442.v3.p2) with the dbGaP controlled-access authorization. The clinical data are available in the NIDDK Central Repository at https://repository.niddk.nih.gov/studies/teddy/. Taxonomic annotation for the MAGs was based on the Genome Taxonomy Database (https://gtdb.ecogenomic.org/; GTDB Release 95). MetaCyc reactions were assigned to proteins based on homology searches against MetaCyc reference proteins in MetaCyc database (https://metacyc.org/). Source data are provided with this paper.

## Source data

Source Data

## Code availability

Codes are freely available under the GNU General Public License v3.0 at https://github.com/thepanlab/Seq2MAG^84^
