# Supplementary material for: Islet autoantibody seroconversion in type-1 diabetes is associated with metagenome-assembled genomes in infant gut microbiomes
Source: Nat Commun. 2022 Jun 21;13:3551. doi: 10.1038/s41467-022-31227-1 (PMC9213500; doi:10.1038/s41467-022-31227-1)
Supplement: Supplementary file 1 — Supplementary Information [file 41467_2022_31227_MOESM1_ESM.pdf]

Islet Autoantibody Seroconversion in Type-1 Diabetes is Associated with Metagenome-Assembled Genomes in Infant Gut Microbiomes

Supplementary information

Supplementary Figure 1. Quality statistics of the metagenome assemblies and MAGs.
Supplementary Figure 2. Functional annotation and clustering of proteins in the metagenome assemblies.
Supplementary Figure 3. Distributions of the core protein families by geographic locations, genders, and delivery modes.
Supplementary Figure 4. Longitudinal abundance profiles of the TEDDY core protein family clusters.
Supplementary Figure 5. Phylogenetic tree of high-quality MAGs.

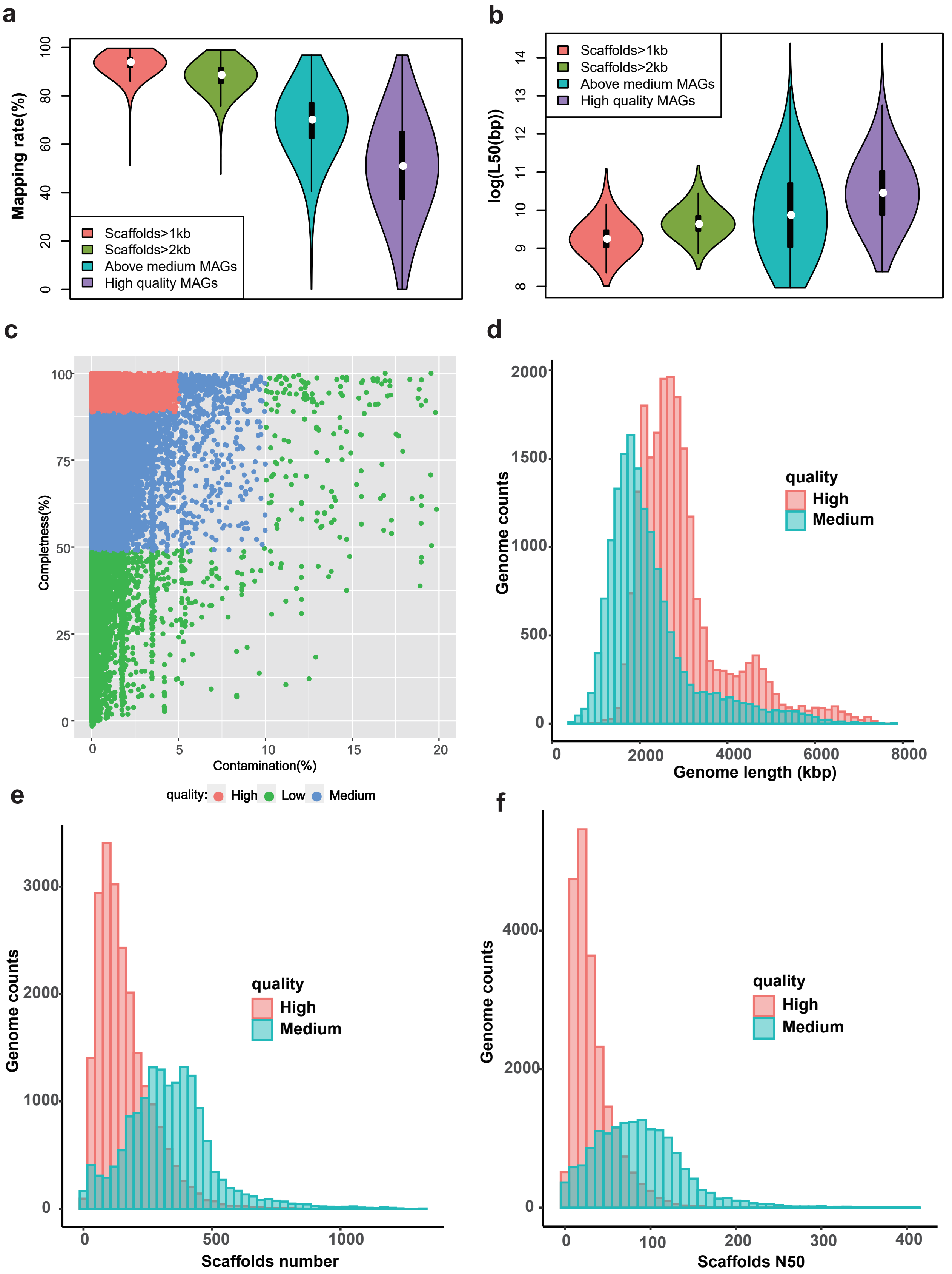

Supplementary Figure 1. Quality statistics of the metagenome assemblies and MAGs. (a) Distribution of the mapping rates across metagenomic sequencing runs (n = 13,245) on the composite subject metagenome assemblies containing scaffolds >1 kb (red violin plot) and scaffolds >2 kb (green violin plot), MAGs of medium and high quality (blue violin plot), and MAGs of high quality only (purple violin plot). **(b)** Distribution of the L50 across subjects for the metagenome assemblies and MAGs {red (n = 887), green (n = 887), blue (n = 37,332), purple (n = 21,536)}. **(c)** Completeness and contamination scores of MAGs of high quality (red dots), medium quality (blue dots), and low quality (red dots). High-quality MAGs have completeness > 90% and contamination < 5%. Medium-quality MAGs have completeness >= 50% and contamination <10%. **(d)** Histogram of the total lengths of the high- and medium-quality MAGs. **(e)** Histogram of the number of scaffolds in the high- and medium-quality MAGs. **(f)** Histogram of the scaffolds N50 in the high- and medium-quality MAGs. Violin plots indicate median (white dot), the first and third quartile (black bar in the center), and the 1.5X interquartile ranges (black lines stretched from the bar). MAG: metagenome-assembled genome, bp: base pair, and kbp: kilobase pair. Source data are provided as a Source Data file.

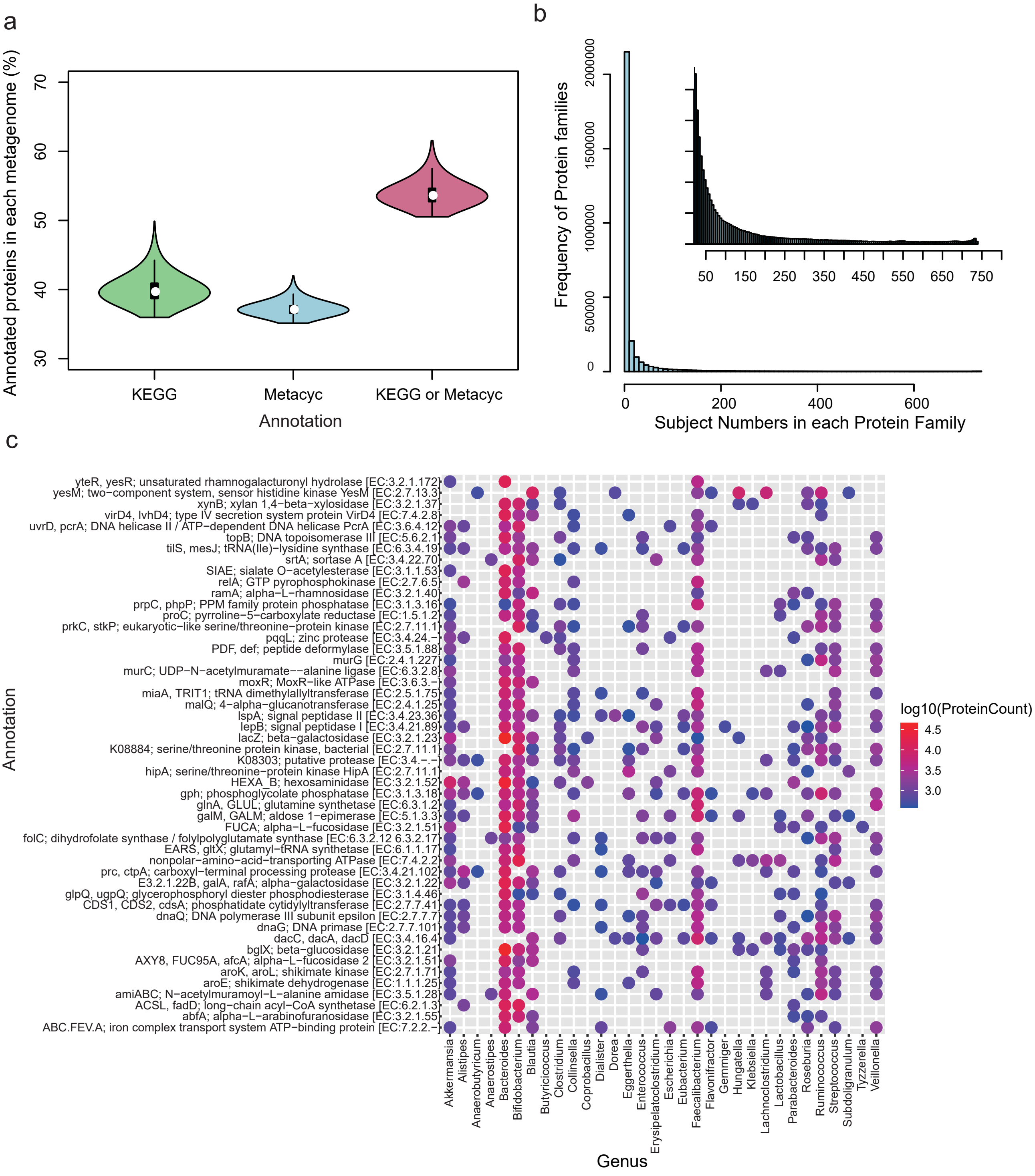

Supplementary Figure 2. Functional annotation and clustering of proteins in the metagenome assemblies. **(a)** Distribution of the percentages of the annotated proteins in all the metagenome assemblies using KEGG and MetaCyc (*n* = 737). Violin plots indicate median (white dot), the first and third quartile (black bar in the center), and the 1.5X interquartile ranges (black lines stretched from the bar). **(b)** Histogram of subject numbers in each protein family. The protein families distributed in more than 50 subjects were shown in a separate histogram. **(c)** Top-50 most frequent E.C. number annotation of the protein families and their genus-level taxonomic distribution. E.C. number: Enzyme Commission number. Source data are provided as a Source Data file.

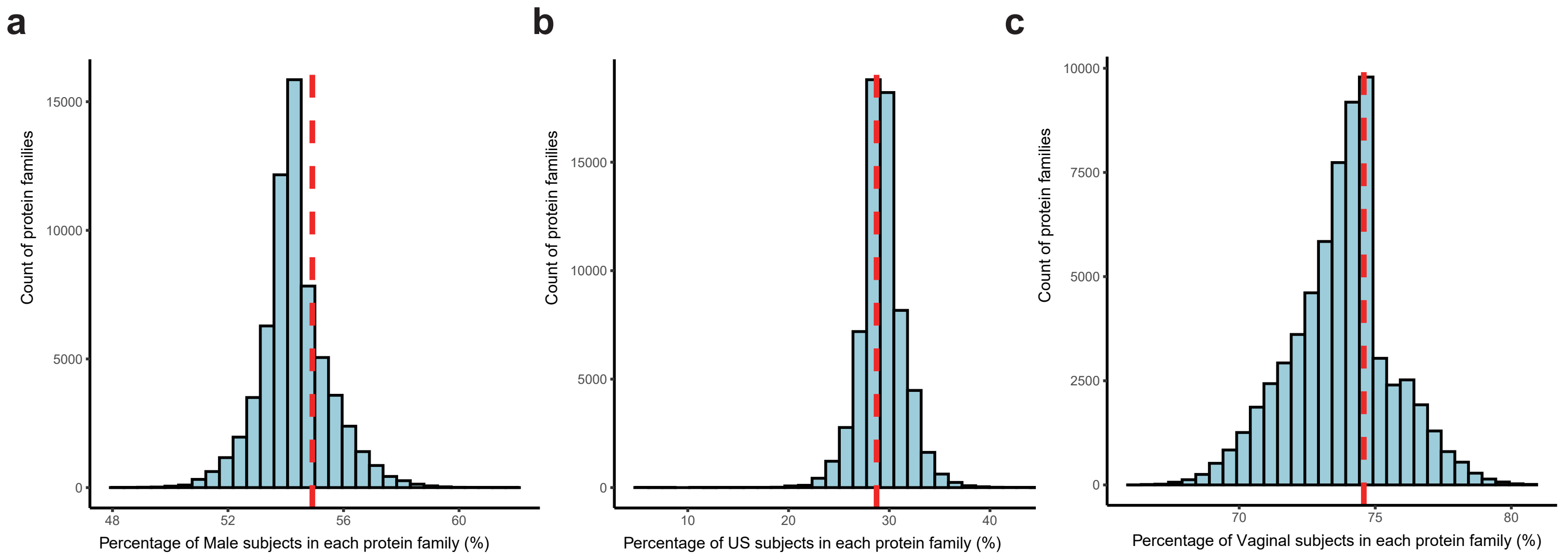

Supplementary Figure 3. Distributions of the core protein families by geographic locations, genders, and delivery modes. (a) Histogram of the percentage of male subjects in each core protein family. The remaining subjects are female. The red line marks the overall percentage of male subjects in the cohort. **(b)** Histogram of the percentage of the subjects in the U.S. in each core protein family. The remaining subjects were located in Europe. The red line marks the overall percentage of the U.S. subject in the cohort. **(c)** Histogram of the percentage of subjects from vaginal delivery in each core protein family. The remaining subjects were delivered by Cesarean section. The red line marks the overall percentage of vaginally delivered subjects in the cohort. Source data are provided as a Source Data file.

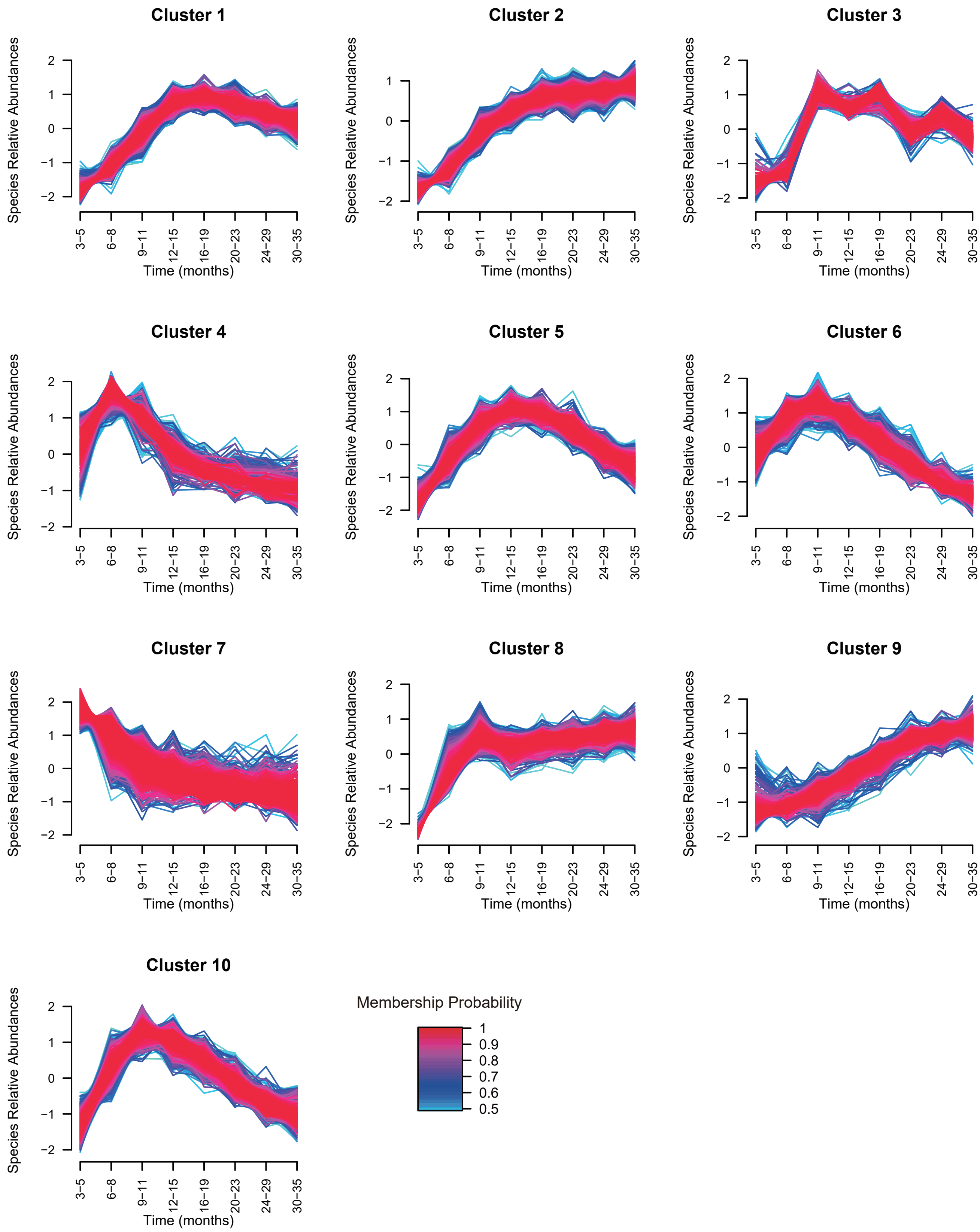

Supplementary Figure 4. Longitudinal abundance profiles of the TEDDY core protein family clusters. The core protein families were clustered into 10 clusters by their average RPKM abundances across the developmental stages, shown in the x-axis. The gene abundances of core protein families were standardized to a mean value of zero and a standard deviation of one. Line colors represent cluster membership probability from 0.5 to 1.0. Core families with membership probabilities below 0.5 are not displayed. TEDDY: The Environmental Determinants of Diabetes in the Young, and RPKM: Reads Per Kilobase per Million reads. Source data are provided as a Source Data file.

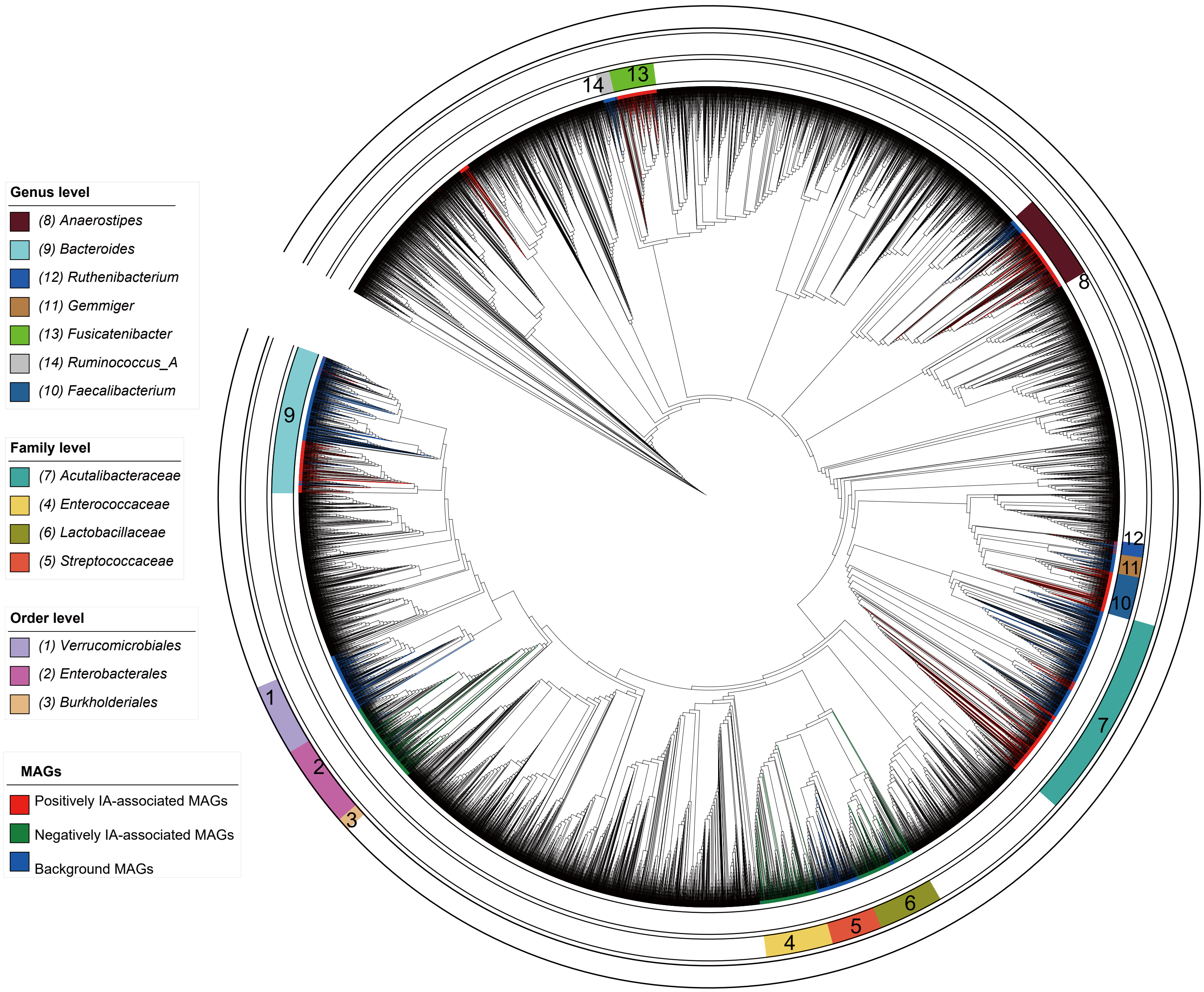

Supplementary Figure 5. Phylogenetic tree of high-quality MAGs. Tree tips correspond to 21,536 high-quality MAGs. The branches containing MAGs with positive IA-association are colored in red and the ones with negative IA-association in green. The branches containing MAGs used as background for comparative genomics are colored in blue. The taxa are highlighted in arcs of varying colors and identified in the legend. MAGs: metagenome-assembled genomes and IA: islet autoantibody. Source data are provided as a Source Data file.
