# Supplementary material for: Islet autoantibody seroconversion in type-1 diabetes is associated with metagenome-assembled genomes in infant gut microbiomes
Source: Nat Commun. 2022 Jun 21;13:3551. doi: 10.1038/s41467-022-31227-1 (PMC9213500; doi:10.1038/s41467-022-31227-1)
Supplement: Supplementary file 2 — Description of Additional Supplementary Files [file 41467_2022_31227_MOESM2_ESM.pdf]

Description of Additional Supplementary Files

File Name: Supplementary Data 1

Description: Summary statistics of the subjectspecific metagenome assemblies.

File Name: Supplementary Data 2

Description: Summary statistics of the high- and medium-quality MAGs.

File Name: Supplementary Data 3

Description: TEDDY microbiome species and representative MAGs.

File Name: Supplementary Data 4

Description: Longitudinal development of human gut microbiota.

File Name: Supplementary Data 5

Description: Core protein families in the TEDDY microbiomes.

File Name: Supplementary Data 6

Description: Enrichment analysis for core protein families. Enricher method in clusterProfiler (two-sided), Benjamini-Hochberg adjusted.

File Name: Supplementary Data 7

Description: Longitudinal development of protein families with KEGG KO annotation.

File Name: Supplementary Data 8

Description: Core protein families with significant association with seroconversion. Generalized linear mixed modeling (two-sided), Benjamini-Hochberg adjusted.

File Name: Supplementary Data 9

Description: MAGs with significant association with seroconversion. Fisher's exact test (one-sided), BenjaminiHochberg adjusted.

File Name: Supplementary Data 10

Description: Summary of the Seroconversion MAGs in species levels.

File Name: Supplementary Data 11

Description: GLMM statistics analysis using species abundance in each taxonomy group to test the association with seroconversion. Generalized linear mixed modeling was used (two-sided), without adjustment.

File Name: Supplementary Data 12

Description: Genomics analysis of LPS biosynthesis pathways comparing between IA-associated Bacteroides MAGs and non-IA-associated Bacteroides MAGs. Wilcoxon test (two-sided) for enrichment analysis and phylogenetic linear modeling (two-sided) for phylogenetic regression analysis, Benjamini-Hochberg adjusted.
