# Supplementary material for: Islet autoantibody seroconversion in type-1 diabetes is associated with metagenome-assembled genomes in infant gut microbiomes
Source: Nat Commun. 2022 Jun 21;13:3551. doi: 10.1038/s41467-022-31227-1 (PMC9213500; doi:10.1038/s41467-022-31227-1)
Supplement: Supplementary file 15 — Reporting Summary [file 41467_2022_31227_MOESM15_ESM.pdf]

nature portfolio

Corresponding author(s): Chongle Pan

Last updated by author(s): May 29, 2022

Reporting Summary

Nature Portfolio wishes to improve the reproducibility of the work that we publish. This form provides structure for consistency and transparency in reporting. For further information on Nature Portfolio policies, see our Editorial Policies and the Editorial Policy Checklist.

Statistics

For all statistical analyses, confirm that the following items are present in the figure legend, table legend, main text, or Methods section.

n/a	Confirmed	
☐	☒	The exact sample size (*n*) for each experimental group/condition, given as a discrete number and unit of measurement
☐	☒	A statement on whether measurements were taken from distinct samples or whether the same sample was measured repeatedly
☐	☒	The statistical test(s) used AND whether they are one- or two-sided<br>*Only common tests should be described solely by name; describe more complex techniques in the Methods section.*
☐	☒	A description of all covariates tested
☐	☒	A description of any assumptions or corrections, such as tests of normality and adjustment for multiple comparisons
☐	☒	A full description of the statistical parameters including central tendency (e.g. means) or other basic estimates (e.g. regression coefficient) AND variation (e.g. standard deviation) or associated estimates of uncertainty (e.g. confidence intervals)
☐	☒	For null hypothesis testing, the test statistic (e.g. *F*, *t*, *r*) with confidence intervals, effect sizes, degrees of freedom and *P* value noted<br>*Give P values as exact values whenever suitable.*
☒	☐	For Bayesian analysis, information on the choice of priors and Markov chain Monte Carlo settings
☒	☐	For hierarchical and complex designs, identification of the appropriate level for tests and full reporting of outcomes
☒	☐	Estimates of effect sizes (e.g. Cohen's *d*, Pearson's *r*), indicating how they were calculated

Our web collection on statistics for biologists contains articles on many of the points above.

Software and code

Policy information about availability of computer code

Data collection	SRA Toolkit tools (version 2.9.6) were used to download SRA data from dbGap and covert raw data from sra format to fastq format.
Data analysis	R v3.6.3; Python v2.7.15 and v3.6.3; SPAdes v3.13.1; MetaBAT 2 v2.12.1; Bowtie 2 v2.3.5.1; pullseq v1.0.2; shrinksam v0.9.0; samtools v0.1.19; CheckM v1.1.2; GTDB-Tk v1.3.0; dRep v2.4.0; PhyloPhlAn 2.0; iTOL v5; Mash v2.2; ANIcalculator v1.0; CD-HIT v4.8.1; Prodigal v2.6.3 ; KofamScan v1.0.0; DIAMOND v0.9.26.127; a series of R packages: glmmTMB v0.2.3, ALDEx2 v1.18.0, Mfuzz v2.54.0, clusterCrit v1.2.8, vegan v2.5-7, phylolm v2.6, clusterProfiler v4.0.5; The scripts have been deposited at https://github.com/thepanlab/Seq2MAG.

For manuscripts utilizing custom algorithms or software that are central to the research but not yet described in published literature, software must be made available to editors and reviewers. We strongly encourage code deposition in a community repository (e.g. GitHub). See the Nature Portfolio guidelines for submitting code & software for further information.

Data

Policy information about availability of data

All manuscripts must include a data availability statement. This statement should provide the following information, where applicable:

- Accession codes, unique identifiers, or web links for publicly available datasets
- A description of any restrictions on data availability
- For clinical datasets or third party data, please ensure that the statement adheres to our policy

All the high-quality MAGs generated in this work have been deposited in the European Nucleotide Archive (ENA) under accession PRJEB40730 (https://www.ebi.ac.uk/ena/browser/view/PRJEB40730). The metagenomic sequencing data and the clinical data are available in NCBI dbGaP under accession phs001442.v3.p2 (https://www.ncbi.nlm.nih.gov/projects/gap/cgi-bin/study.cgi?study_id=phs001442.v3.p2), in accordance with the dbGaP controlled-access authorization process and NIDDK Central Repository website at https://repository.niddk.nih.gov/studies/teddy/. Taxonomic annotation for the MAGs was based on

the Genome Taxonomy Database (https://gtdb.ecogenomic.org/; GTDB Release 95). Metacyc reactions were assigned to proteins based on homology searches against MetaCyc reference proteins in MetaCyc database (https://metacyc.org/). Source Data are provided with this paper.

Field-specific reporting

Please select the one below that is the best fit for your research. If you are not sure, read the appropriate sections before making your selection.

[x] Life sciences   [ ] Behavioural & social sciences   [ ] Ecological, evolutionary & environmental sciences

For a reference copy of the document with all sections, see nature.com/documents/nr-reporting-summary-flat.pdf

Life sciences study design

All studies must disclose on these points even when the disclosure is negative.

Sample size	No statistical method was used to pre-determine sample size. We included all the metagenomic sequencing samples obtained from dbGaP phs001442.v3.p2. The metagenome assembly and binning were performed using metagenomic data from a time-course collection of 12,276 fecal samples from 887 subjects. The statistical comparison of the islet autoimmunity (IA) cases versus the controls was conducted using metagenomic abundance information in 10,584 fecal samples from 660 subjects who donated at least 4 samples and have clinical information, including IA abundances portending seroconversion, available at matched timepoints.
Data exclusions	There were no specific pre-established exclusion criteria. In the statistical comparison of the islet autoimmunity (IA) cases versus the controls, we excluded 227 subjects who have no clinical information on their IA seroconversion status or donated less than 4 fecal samples, because these subjects have an unknown IA fixed effect or an insufficient number of time-points in the generalized linear mixed models.
Replication	The analysis was done on an observational cohort without replication.
Randomization	Randomization was not used in this study, as this is a re-analysis of a controlled-access metagenomic data from the TEDDY study.
Blinding	Blinding was not used, as this is a re-analysis of a controlled-access metagenomic data from the TEDDY study.

Reporting for specific materials, systems and methods

We require information from authors about some types of materials, experimental systems and methods used in many studies. Here, indicate whether each material, system or method listed is relevant to your study. If you are not sure if a list item applies to your research, read the appropriate section before selecting a response.

Materials & experimental systems

n/a	Involved in the study	
[x]	[ ]	Antibodies
[x]	[ ]	Eukaryotic cell lines
[x]	[ ]	Palaeontology and archaeology
[x]	[ ]	Animals and other organisms
[x]	[ ]	Human research participants
[x]	[ ]	Clinical data
[x]	[ ]	Dual use research of concern

Methods

n/a	Involved in the study	
[x]	[ ]	ChIP-seq
[x]	[ ]	Flow cytometry
[x]	[ ]	MRI-based neuroimaging
